# Supplementary material for: JASPer controls interphase histone H3S10 phosphorylation by chromosomal kinase JIL-1 in Drosophila
Source: Nat Commun. 2019 Nov 25;10:5343. doi: 10.1038/s41467-019-13174-6 (PMC6877644; doi:10.1038/s41467-019-13174-6)
Supplement: Supplementary file 3 — Description of Additional Supplementary Files [file 41467_2019_13174_MOESM3_ESM.pdf]

Description of Additional Supplementary Files

File Name: Supplementary Data 1

Description: The JASPer interaction network. Table of complete IP-MS data presented in figure 7 reporting mean log2 fold enrichment in α-JASPer IP from nuclear extract from fly embryos (n = 6) versus control IP (n = 5) and associated p-values.
