# Supplementary material for: JASPer controls interphase histone H3S10 phosphorylation by chromosomal kinase JIL-1 in Drosophila
Source: Nat Commun. 2019 Nov 25;10:5343. doi: 10.1038/s41467-019-13174-6 (PMC6877644; doi:10.1038/s41467-019-13174-6)
Supplement: Supplementary file 5 — Reporting Summary [file 41467_2019_13174_MOESM5_ESM.pdf]

nature research

Corresponding author(s): Catherine Regnard
Last updated by author(s): 10/02/2019

Reporting Summary

Nature Research wishes to improve the reproducibility of the work that we publish. This form provides structure for consistency and transparency in reporting. For further information on Nature Research policies, see Authors & Referees and the Editorial Policy Checklist.

Statistics

For all statistical analyses, confirm that the following items are present in the figure legend, table legend, main text, or Methods section.

n/a	Confirmed	
☐	☒	The exact sample size (*n*) for each experimental group/condition, given as a discrete number and unit of measurement
☐	☒	A statement on whether measurements were taken from distinct samples or whether the same sample was measured repeatedly
☐	☒	The statistical test(s) used AND whether they are one- or two-sided<br>*Only common tests should be described solely by name; describe more complex techniques in the Methods section.*
☒	☐	A description of all covariates tested
☐	☒	A description of any assumptions or corrections, such as tests of normality and adjustment for multiple comparisons
☐	☒	A full description of the statistical parameters including central tendency (e.g. means) or other basic estimates (e.g. regression coefficient) AND variation (e.g. standard deviation) or associated estimates of uncertainty (e.g. confidence intervals)
☐	☒	For null hypothesis testing, the test statistic (e.g. *F*, *t*, *r*) with confidence intervals, effect sizes, degrees of freedom and *P* value noted<br>*Give P values as exact values whenever suitable.*
☒	☐	For Bayesian analysis, information on the choice of priors and Markov chain Monte Carlo settings
☒	☐	For hierarchical and complex designs, identification of the appropriate level for tests and full reporting of outcomes
☐	☒	Estimates of effect sizes (e.g. Cohen's *d*, Pearson's *r*), indicating how they were calculated

Our web collection on statistics for biologists contains articles on many of the points above.

Software and code

Policy information about availability of computer code

Data collection	RNA-seq and ChIP-seq libraries were sequenced with Illumina HiSeq1500 at LAFUGA. MS data was collected with Thermo Fischer Qexactive HF and proteins were identified and quantified with MaxQuant version 1.5.2.8 .
Data analysis	General Software: R version 3.5.1 and Bioconductor version 3.8.<br>Sequencing data was analyzed with: SAMtools version 1.3.1, BEDtools version 2.26.0, Bowtie version 1.1.2, STAR version 2.6.0, RSEM version 1.3.0, DESeq2 version 1.22.1, tsTools version 0.1.1.<br>MS data was analyzed with: DEP version 1.4.0 (R), MSnbase version 2.8.1 (R), PANTHER, Cytoscape version 3.7.0 with STRING database.

For manuscripts utilizing custom algorithms or software that are central to the research but not yet described in published literature, software must be made available to editors/reviewers. We strongly encourage code deposition in a community repository (e.g. GitHub). See the Nature Research guidelines for submitting code & software for further information.

Data

Policy information about availability of data

All manuscripts must include a data availability statement. This statement should provide the following information, where applicable:

- Accession codes, unique identifiers, or web links for publicly available datasets
- A list of figures that have associated raw data
- A description of any restrictions on data availability

Accession codes: All sequencing data are available at https://www.ncbi.nlm.nih.gov/geo/query/acc.cgi?acc=GSE128457, secure token 'crsbcauunxsvlyv'
Mass spectrometry results are available at ProteomeXchange Consortium with the dataset identifier PXD012790, username 'reviewer92166@ebi.ac.uk' and password '4TDSJ7If'
List of figures that have associated raw data: Fig.3d, e; Fig.4 a, b, c; Fig.5a, b;Fig. 6a, b, c; Fig.7.
There is no restriction in data availability.

Field-specific reporting

Please select the one below that is the best fit for your research. If you are not sure, read the appropriate sections before making your selection.

☒ Life sciences ☐ Behavioural & social sciences ☐ Ecological, evolutionary & environmental sciences

For a reference copy of the document with all sections, see nature.com/documents/nr-reporting-summary-flat.pdf

Life sciences study design

All studies must disclose on these points even when the disclosure is negative.

Sample size	Sample sizes were not determined by statistical test. Generally, => 3 biological replicates were used for ChIP-seq and => 4 for RNA-seq, based on community standards.
Data exclusions	RNA-seq samples with low number of reads were excluded.
Replication	All ChIP replicates for each antibody have been prepared on independent Chromatin preparations, indicated by the number of replicates (n). When possible different antibodies against one antigen (JIL-1, JASPer) have been used (in general 3 independent replicates). The libraries of biological replicates have been prepared and sequenced together or separately. The RNAi experiments for ChIP-seq analysis have been done with 2 independent dsRNA constructs for each target knock down as described in the methods.<br><br>The RNAi experiments for RNA-seq analysis have been done with 2 independent dsRNA constructs for each target knock down as described in the methods. Biological replicates have been prepared following the same protocol in different weeks. For each set of biological replicates libraries have been prepared together. Sequencing were done together or/and separately.
Randomization	Not applicable
Blinding	Not applicable

Reporting for specific materials, systems and methods

We require information from authors about some types of materials, experimental systems and methods used in many studies. Here, indicate whether each material, system or method listed is relevant to your study. If you are not sure if a list item applies to your research, read the appropriate section before selecting a response.

Materials & experimental systems

n/a	Involved in the study	
☐	☒	Antibodies
☐	☒	Eukaryotic cell lines
☒	☐	Palaeontology
☒	☐	Animals and other organisms
☒	☐	Human research participants
☒	☐	Clinical data

Methods

n/a	Involved in the study	
☐	☒	ChIP-seq
☒	☐	Flow cytometry
☒	☐	MRI-based neuroimaging

Antibodies

Antibodies used	The list of all used antibodies is in the manuscript. Lot numbers of commercial antibodies used for ChIP were added in the corresponding section.
Validation	Commercial antibodies were validated by the corresponding distributor and published antibodies in the corresponding publication. Novel antibodies were tested for specificity by knock down experiments of the corresponding antigen.

Eukaryotic cell lines

Policy information about cell lines

Cell line source(s)	The drosophila cell lines have been ordered from the Drosophila Genomics Resource Center. The S2 cells (Stock #181) are from April 2014 and the Kc167 cells (Stock#1) from July 2014.
Authentication	S2 and Kc cells were authenticated a posteriori as male and female cell lines according to their X chromosomal DNA content in the input DNA from Chromatin samples (Fig. S7, c, d, e) and in other experiments done in the laboratory (e.g. immunostaining for DCC subunits at the X territory).

S2 DRSC (DGRC stock # 181), Kc167 (DGRC stock # 1), D. virilis 79f7Dv3 cells, Sf21 cells (Thermo Fischer).

Mycoplasma contamination

The cell lines used in this study have been tested negative for mycoplasma contamination upon arrival and along the time frame of our study.

Commonly misidentified lines
(See ICLAC register)

None used.

ChIP-seq

Data deposition

☒ Confirm that both raw and final processed data have been deposited in a public database such as GEO.

☒ Confirm that you have deposited or provided access to graph files (e.g. BED files) for the called peaks.

Data access links
May remain private before publication.

All sequencing data are available at https://www.ncbi.nlm.nih.gov/geo/query/acc.cgi?acc=GSE128457

Files in database submission

ChIPseq.kc.jasper_1.fastqsanger.gz
ChIPseq.kc.jasper_2.fastqsanger.gz
ChIPseq.kc.jasper_3.fastqsanger.gz
ChIPseq.s2.jasper_1.fastqsanger.gz
ChIPseq.s2.jasper_2.fastqsanger.gz
ChIPseq.s2.jasper_3.fastqsanger.gz
ChIPseq.s2.jasper_4.fastqsanger.gz
ChIPseq.kc.jasper_1.norm.bw
ChIPseq.kc.jasper_2.norm.bw
ChIPseq.kc.jasper_3.norm.bw
ChIPseq.s2.jasper_1.norm.bw
ChIPseq.s2.jasper_2.norm.bw
ChIPseq.s2.jasper_3.norm.bw
ChIPseq.s2.jasper_4.norm.bw
ChIP_JASPer_gst_RNAi_2.fastqsanger.gz
ChIP_JASPer_gst_RNAi_3.fastqsanger.gz
ChIP_JASPer_gst_RNAi_2.norm.bw
ChIP_JASPer_gst_RNAi_3.norm.bw
RNAseq.kc.jasper1.batch1_1.fastqsanger.gz
RNAseq.kc.jasper1.batch1_2.fastqsanger.gz
RNAseq.kc.jasper2.batch1_1.fastqsanger.gz
RNAseq.kc.jasper2.batch1_2.fastqsanger.gz
RNAseq.kc.jasper3.batch2_1.fastqsanger.gz
RNAseq.kc.jasper3.batch2_2.fastqsanger.gz
RNAseq.kc.jasper4.batch2_1.fastqsanger.gz
RNAseq.kc.jasper4.batch2_2.fastqsanger.gz
RNAseq.s2.jasper4.batch1_1.fastqsanger.gz
RNAseq.s2.jasper4.batch1_2.fastqsanger.gz
RNAseq.s2.jasper5.batch1_1.fastqsanger.gz
RNAseq.s2.jasper5.batch1_2.fastqsanger.gz
RNAseq.s2.jasper8.batch2_1.fastqsanger.gz
RNAseq.s2.jasper8.batch2_2.fastqsanger.gz
RNAseq.s2.jasper9.batch2_1.fastqsanger.gz
RNAseq.s2.jasper9.batch2_2.fastqsanger.gz
RNAseq.kc.jasper1.batch1.ReadsPerGene.out.tab
RNAseq.kc.jasper2.batch1.ReadsPerGene.out.tab
RNAseq.kc.jasper3.batch2.ReadsPerGene.out.tab
RNAseq.kc.jasper4.batch2.ReadsPerGene.out.tab
RNAseq.s2.jasper4.batch1.ReadsPerGene.out.tab
RNAseq.s2.jasper5.batch1.ReadsPerGene.out.tab
RNAseq.s2.jasper8.batch2.ReadsPerGene.out.tab
RNAseq.s2.jasper9.batch2.ReadsPerGene.out.tab
ChIPseq.s2.h3k9me2_gfp_1.fastq.gz
ChIPseq.s2.h3k9me2_gst_2.fastq.gz
ChIPseq.s2.h3k9me2_gst_3.fastq.gz
ChIPseq.s2.h3k9me2_jil1_1.fastq.gz
ChIPseq.s2.h3k9me2_jil2_2.fastq.gz
ChIPseq.s2.h3k9me2_jil2_3.fastq.gz
ChIPseq.s2.h4k16ac_gfp_1.fastq.gz
ChIPseq.s2.h4k16ac_gst_2.fastq.gz
ChIPseq.s2.h4k16ac_gst_3.fastq.gz
ChIPseq.s2.h4k16ac_jil1_1.fastq.gz
ChIPseq.s2.h4k16ac_jil2_2.fastq.gz
ChIPseq.s2.h4k16ac_jil2_3.fastq.gz

ChIPseq.s2.input_gfp_1.fastq.gz
ChIPseq.s2.input_gst_2.fastq.gz
ChIPseq.s2.input_gst_3.fastq.gz
ChIPseq.s2.input_jil1_1.fastq.gz
ChIPseq.s2.input_jil2_2.fastq.gz
ChIPseq.s2.input_jil2_3.fastq.gz
ChIPseq.s2.jasper_gfp_1.fastq.gz
ChIPseq.s2.jasper_gst_2.fastq.gz
ChIPseq.s2.jasper_gst_3.fastq.gz
ChIPseq.s2.jasper_jil1_1.fastq.gz
ChIPseq.s2.jasper_jil2_2.fastq.gz
ChIPseq.s2.jasper_jil2_3.fastq.gz
ChIPseq.s2.msl3_gst_2.fastq.gz
ChIPseq.s2.msl3_gst_3.fastq.gz
ChIPseq.s2.msl3_jil2_2.fastq.gz
ChIPseq.s2.msl3_jil2_3.fastq.gz
ChIPseq.s2.h3k9me2_gfp_1.norm.bw
ChIPseq.s2.h3k9me2_gst_2.norm.bw
ChIPseq.s2.h3k9me2_gst_3.norm.bw
ChIPseq.s2.h3k9me2_jil1_1.norm.bw
ChIPseq.s2.h3k9me2_jil2_2.norm.bw
ChIPseq.s2.h3k9me2_jil2_3.norm.bw
ChIPseq.s2.h4k16ac_gfp_1.norm.bw
ChIPseq.s2.h4k16ac_gst_2.norm.bw
ChIPseq.s2.h4k16ac_gst_3.norm.bw
ChIPseq.s2.h4k16ac_jil1_1.norm.bw
ChIPseq.s2.h4k16ac_jil2_2.normDvir.norm.bw
ChIPseq.s2.h4k16ac_jil2_3.norm.bw
ChIPseq.s2.input_gfp_1.cov.bw
ChIPseq.s2.input_gst_2.cov.bw
ChIPseq.s2.input_gst_3.cov.bw
ChIPseq.s2.input_jil1_1.cov.bw
ChIPseq.s2.input_jil2_2.cov.bw
ChIPseq.s2.input_jil2_3.cov.bw
ChIPseq.s2.jasper_gfp_1.norm.bw
ChIPseq.s2.jasper_gst_2.norm.bw
ChIPseq.s2.jasper_gst_3.norm.bw
ChIPseq.s2.jasper_jil1_1.norm.bw
ChIPseq.s2.jasper_jil2_2.norm.bw
ChIPseq.s2.jasper_jil2_3.norm.bw
ChIPseq.s2.msl3_gst_2.norm.bw
ChIPseq.s2.msl3_gst_3.normDvir.norm.bw
ChIPseq.s2.msl3_jil2_2.norm.bw
ChIPseq.s2.msl3_jil2_3.norm.bw
ChIPseq.kc.h3k36me3_3.fastqsanger.gz
ChIPseq.kc.input_3.fastqsanger.gz
ChIPseq.kc.msl3_1.fastqsanger.gz
ChIPseq.kc.msl3_2.fastqsanger.gz
ChIPseq.kc.msl3_3.fastqsanger.gz
ChIPseq.s2.h3k36me3_3.fastqsanger.gz
ChIPseq.s2.h3k36me3_4.fastqsanger.gz
ChIPseq.s2.input_3.fastqsanger.gz
ChIPseq.s2.input_4.fastqsanger.gz
ChIPseq.s2.input_5.fastqsanger.gz
ChIPseq.s2.input_6.fastqsanger.gz
ChIPseq.s2.input_7.fastqsanger.gz
ChIPseq.s2.jil_3.fastqsanger.gz
ChIPseq.s2.jil_4.fastqsanger.gz
ChIPseq.s2.jil_5_R69AP.fastqsanger.gz
ChIPseq.s2.jil_5_R69S.fastqsanger.gz
ChIPseq.s2.jil_5_hope.fastqsanger.gz
ChIPseq.s2.msl3_2.fastqsanger.gz
ChIPseq.s2.msl3_6.fastqsanger.gz
ChIPseq.s2.msl3_7.fastqsanger.gz
ChIPseq.kc.h3k36me3_3.norm.bw
ChIPseq.kc.input_3_cov.bw
ChIPseq.kc.msl3_1.norm.bw
ChIPseq.kc.msl3_2.norm.bw
ChIPseq.kc.msl3_3.norm.bw
ChIPseq.s2.h3k36me3_3.norm.bw
ChIPseq.s2.h3k36me3_4.norm.bw
ChIPseq.s2.input_3_cov.bw
ChIPseq.s2.input_4_cov.bw
ChIPseq.s2.input_5_cov.bw
ChIPseq.s2.input_6_cov.bw

ChIPseq.s2.input_7_cov.bw
ChIPseq.s2.jil_3.norm.bw
ChIPseq.s2.jil_4.norm.bw
ChIPseq.s2.jil_5_R69AP.norm.bw
ChIPseq.s2.jil_5_R69S.norm.bw
ChIPseq.s2.jil_5_hope.norm.bw
ChIPseq.s2.msl3_2.norm.bw
ChIPseq.s2.msl3_6.norm.bw
ChIPseq.s2.msl3_7.norm.bw
RNAseq.kc.ctrl1.batch1_1.fastqsanger.gz
RNAseq.kc.ctrl1.batch1_2.fastqsanger.gz
RNAseq.kc.ctrl2.batch1_1.fastqsanger.gz
RNAseq.kc.ctrl2.batch1_2.fastqsanger.gz
RNAseq.kc.ctrl3.batch2_1.fastqsanger.gz
RNAseq.kc.ctrl3.batch2_2.fastqsanger.gz
RNAseq.kc.ctrl4.batch2_1.fastqsanger.gz
RNAseq.kc.ctrl4.batch2_2.fastqsanger.gz
RNAseq.kc.jil1.batch1_1.fastqsanger.gz
RNAseq.kc.jil1.batch1_2.fastqsanger.gz
RNAseq.kc.jil2.batch1_1.fastqsanger.gz
RNAseq.kc.jil2.batch1_2.fastqsanger.gz
RNAseq.kc.jil3.batch2_1.fastqsanger.gz
RNAseq.kc.jil3.batch2_2.fastqsanger.gz
RNAseq.kc.jil4.batch2_1.fastqsanger.gz
RNAseq.kc.jil4.batch2_2.fastqsanger.gz
RNAseq.s2.ctrl11.batch3_1.fastqsanger.gz
RNAseq.s2.ctrl11.batch3_2.fastqsanger.gz
RNAseq.s2.ctrl4.batch1_1.fastqsanger.gz
RNAseq.s2.ctrl4.batch1_2.fastqsanger.gz
RNAseq.s2.ctrl5.batch1_1.fastqsanger.gz
RNAseq.s2.ctrl5.batch1_2.fastqsanger.gz
RNAseq.s2.ctrl8.batch2_1.fastqsanger.gz
RNAseq.s2.ctrl8.batch2_2.fastqsanger.gz
RNAseq.s2.ctrl9.batch2_1.fastqsanger.gz
RNAseq.s2.ctrl9.batch2_2.fastqsanger.gz
RNAseq.s2.jil11.batch3_1.fastqsanger.gz
RNAseq.s2.jil11.batch3_2.fastqsanger.gz
RNAseq.s2.jil4.batch1_1.fastqsanger.gz
RNAseq.s2.jil4.batch1_2.fastqsanger.gz
RNAseq.s2.jil5.batch1_1.fastqsanger.gz
RNAseq.s2.jil5.batch1_2.fastqsanger.gz
RNAseq.s2.jil8.batch2_1.fastqsanger.gz
RNAseq.s2.jil8.batch2_2.fastqsanger.gz
RNAseq.s2.jil9.batch2_1.fastqsanger.gz
RNAseq.s2.jil9.batch2_2.fastqsanger.gz
RNAseq.kc.ctrl1.batch1.ReadsPerGene.out.tab
RNAseq.kc.ctrl2.batch1.ReadsPerGene.out.tab
RNAseq.kc.ctrl3.batch2.ReadsPerGene.out.tab
RNAseq.kc.ctrl4.batch2.ReadsPerGene.out.tab
RNAseq.kc.jil1.batch1.ReadsPerGene.out.tab
RNAseq.kc.jil2.batch1.ReadsPerGene.out.tab
RNAseq.kc.jil3.batch2.ReadsPerGene.out.tab
RNAseq.kc.jil4.batch2.ReadsPerGene.out.tab
RNAseq.s2.ctrl11.batch3.ReadsPerGene.out.tab
RNAseq.s2.ctrl4.batch1.ReadsPerGene.out.tab
RNAseq.s2.ctrl5.batch1.ReadsPerGene.out.tab
RNAseq.s2.ctrl8.batch2.ReadsPerGene.out.tab
RNAseq.s2.ctrl9.batch2.ReadsPerGene.out.tab
RNAseq.s2.jil11.batch3.ReadsPerGene.out.tab
RNAseq.s2.jil4.batch1.ReadsPerGene.out.tab
RNAseq.s2.jil5.batch1.ReadsPerGene.out.tab
RNAseq.s2.jil8.batch2.ReadsPerGene.out.tab
RNAseq.s2.jil9.batch2.ReadsPerGene.out.tab

Genome browser session
(e.g. UCSC)

bigwig files in BDGP6 genome annotation for visualization can be accessed at https://www.ncbi.nlm.nih.gov/geo/query/acc.cgi?acc=GSE128457

Methodology

Replicates

Replicates agreement is illustrated in Fig. S5 and was analyzed by hierarchical clustering and Pearson correlation coefficient.

Sequencing depth

Generally, 20-25 Mio. 50bp single-end reads for ChIP-seq and 15-50 Mio. 50bp paired-end reads for RNA-seq on average.

Antibodies	α-H3K36me3 (Abcam, ab9050, Lot:GR56602-1), α-H3K9me2 (Abcam, ab1220, Lot:GR3228498-2), α-H4K16ac (Millipore, 07-329, Lot:2960455)
Peak calling parameters	NA
Data quality	Replicates agreement was analyzed by hierarchical clustering and Pearson correlation coefficient.
Software	R version 3.5.1
